# Shared Care and GP Records: Implications for Patient Safety

**DOI:** 10.1192/bjo.2026.11483

**Published:** 2026-06-30

**Authors:** Aneal Sidhu, Soham De, Abhinav Rastogi

**Affiliations:** Midlands Partnership University NHS Foundation Trust, Telford, United Kingdom

## Abstract

**Aims:**

GP connect is an NHS digital service that allows approved professionals to access patient GP records and provides essential information such as their current medication. Information relating to current medication is vital for clinical decisions and patient safety, particularly when patients present to a new service such as A&E. The clinical risks associated with a lack of information can be even more significant when medications such as clozapine, lithiumand other antipsychotics are being prescribed.

Increasingly, secondary care services are prescribing various psychotropics under shared care arrangements. This QI project sets out to assess the accuracy of GP records in noting shared care medications, barriers to recording these and potential solutions. Several primary care services are relying on AI tools to summarise letters and both opportunities and threats relating to this are discussed.

**Methods:**

120 records were reviewed for patients under a community mental health team in Telford, Shropshire. These patients constituted one consultant’s case load.

Each patient’s psychotropic medication on their GP connect medication list was reviewed against the most recent correspondence sent to the GP by secondary care.

**Results:**

55 of 120 patients had an inaccurate medication record on GP Connect, constituting a total of 77 inaccurately recorded psychotropic medications. Secondary care was the prescriber for 75 of these medications. Out of the 77, there were 55 psychotropics which were not recorded at all. The remaining inaccuracies included inaccurate doses or showing a discontinued psychotropic as a current repeat. The clozapinestatus of 7 patients and lithium status of 2 were not recorded on GP Connect, and 16 depot medications were missing.

Liaison with primary care, including the pharmacy team, was done to identify solutions to rectify these inaccuracies in the GP records, and to develop processes to ensure they are aligned to include secondary care prescribing.

**Conclusion:**

This QI project evidences significant challenges with prescribing across services and access to uniform information. This can have serious implications on patient safety particularly in medical emergencies. In the current climate with pressures on both primary and secondary care, closer liaison with primary care that goes beyond sharing correspondence is needed, including development of joint pathways. Several GP practices are utilising AI tools to review incoming correspondence which can be useful, however agreed protocols are required to facilitate this.

